# Spatial and temporal patterns of SARS-CoV-2 infection in uMgungundlovu, KwaZulu-Natal, South Africa

**DOI:** 10.1371/journal.pone.0317648

**Published:** 2026-04-15

**Authors:** Radiya Gangat, Veranyuy Ngah, Rushambwa Tawonga, Justine I. Blanford, Jabulani Ronnie Ncayiyana, Peter Suwirakwenda Nyasulu

**Affiliations:** 1 Division of Epidemiology and Biostatistics, Department of Global Health, Faculty of Medicine and Health Sciences, Stellenbosch University, Cape Town, Western Cape, South Africa; 2 Department of Health, uMgungundlovu Health District Office, Pietermaritzburg, KwaZulu-Natal, South Africa; 3 School of Development Studies, University of KwaZulu-Natal, Durban, KwaZulu-Natal, South Africa; 4 Department of Earth Observation Science (EOS), Faculty of Geo-Information Science and Earth Observation (ITC), University of Twente, Enschede, Netherlands; 5 Division of Health Measurement Sciences, School of Health Systems and Public Health, Faculty of Health Sciences, University of Pretoria, Pretoria, Gauteng, South Africa; 6 Division of Epidemiology and Biostatistics, School of Public Health, Faculty of Health Sciences, University of the Witwatersrand, Johannesburg, South Africa; Environmental Research Center (CRE), ALGERIA

## Abstract

**Background:**

Investigating the spatial distribution of SARS-CoV-2 at a local level and describing the pattern of disease occurrence can be used as the basis for efficient prevention and control measures. This research project aims to utilize geospatial analysis to understand the distribution patterns of SARS-CoV-2 infection and its relationship with certain co-existing factors in uMgungundlovu district, KwaZulu-Natal.

**Methods:**

Spatial characteristics of SARS-CoV-2 were investigated over the first four waves of transmission using ESRI ArcGIS Pro v2.0, including Local Indicators of Spatial Association (LISA) with Moran’s “I” as the measure of spatial autocorrelation; and Kernel Density Estimation (KDE). In implementing temporal analysis, time series analysis using the Python Seaborn library was used, with separate modelling carried out for each wave.

**Results:**

Statistically significant SARS-CoV-2 incidence rates were noted across age groups with p-values = 0.0000. Statistically significant clustering was evident in wave 1 and wave 3 (Moran’s I respectively: wave 1–0.096; wave 2–0.023; wave 3–0.039; wave 4–0.023). The KDE (Highest density of cases: wave 1: 25.0001–50.0, wave 2: 25.0001–50.0, wave 3: 100.001–150.0, wave 4: 50.0001–100.0). Temporal analysis showed more fluctuation at the beginning of each wave with less fluctuation in daily identified cases within the middle to end of each wave.

**Conclusion:**

A Geospatial approach of analysing infectious disease transmission is proposed to guide control efforts (e.g., testing/tracing and vaccine rollout) for populations at higher vulnerability. Additionally, the nature and configuration of the social and built environment may be associated with increased infection. However, locally specific empirical research is required to assess other relevant factors associated with increased infection.

## Introduction

In 2019 a novel coronavirus disease was identified with cases being epidemiologically linked to a wet market in Wuhan, China; the virus was later named SARS-CoV-2 with the resulting disease named COVID-19. This respiratory droplet or close contact transmitted viral outbreak quickly reached pandemic status with rapid infection and a high mortality rate, becoming a global health emergency [1]. As of October 4, 2023, the World Health Organization recorded 771 151 224 confirmed cases and 6,960,783 deaths [2].

By the end of January, 2022, South Africa experienced four recognisable SARS-CoV-2 pandemic waves with approximately 3-month periods of low transmission between each wave, each wave was dominated by one variant of concern: the ancestral strain with an Asp614Gly mutation during the first wave, the beta variant (B.1.351) during the second wave, the delta variant (B.1.617.2) during the third wave, and the omicron variant (B.1.1.529) during the fourth wave [3]. The first reported South African case was announced on March 5, 2020, residing within uMgungundlovu district in the local municipality of uMngeni. Additionally, uMgungundlovu district has experienced the second-highest incidence rate in the province [4]. However, the impact of infection within the district has not been well documented with little available information and many unknowns.

The SARS-CoV-2 global spread highlighted spatial characteristics; geographic differences have affected the spatial distribution of cases [2,3]. Geographic Information Science (GIS) can be useful in research related to geography, space and other spatio-temporal factors [4]. Research needs include crossing different variables to interpret the transmission of SARS-CoV-2 including spatial analysis and spatiotemporal dimensions; geographical impact on public health measures; and predictive modelling of the evolution of the disease. Thus, using geospatial and statistical tools has become particularly relevant in the interpretation of the SARS-CoV-2 pandemic [5].

Globally, the spatial distribution of infection has been widespread, however, a distinct asymmetry has been observed in terms of incidence rates and distribution of cases according to geographical location [6]. With emerging pathogenic outbreaks, it is critical to understand the dynamics of infection transmission. This is of particular importance when analysing the contagion over time and projecting the future epidemiological situation. Local research related to the dynamics of infection transmission including details related to incidence rates and distribution of cases are largely unknown in the study setting making it difficult to project future epidemiological situations. In this study, Geographic information systems (GIS) were used to analyse the spatial distribution patterns of SARS-CoV-2 in uMgungundlovu district, KwaZulu-Natal Province, South Africa, and to highlight the hot spot and cold spot areas of high and low infection respectively.

## Materials and methods

### Study design and participants

Cross-sectional study design: all laboratory confirmed SARS-CoV-2 positive individuals residing within uMgungundlovu district during each of the first four waves experienced formed part of the study population.

### Study setting

**Fig 1:**
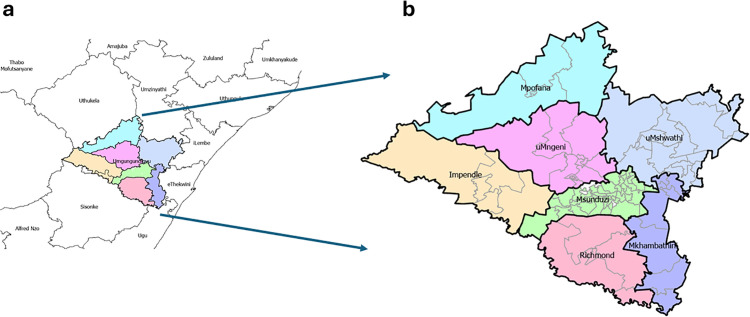
The (a) KwaZulu-Natal (KZN) Province where the (b) district of uMgungundlovu is located. Boundary data are from esri (S3 Table) and background map provided by Esri (Esri South Africa, TomTom, Garmin, FAO, METI/NASA, USGS, CGIAR). Maps were created using ArcGIS Pro software by Esri.

The district of uMgungundlovu lies in the interior of KwaZulu-Natal (KZN) Province; uMgungundlovu district borders Ethekwini, Ilembe, uMzinyathi, uThukela, Harry Gwala and Ugu districts within KwaZulu-Natal, uMgungundlovu district also borders the country of Lesotho (Fig 1). The district provides health care services to 10% of the KZN population estimated at 1 017 763 [7]. uMgungundlovu covers an area of 9189 square kilometres and is comprised of 7 local municipalities; these include uMsunduzi, Impendle, Richmond, uMngeni, uMkhambathini, uMshwathi and Mpofana; a description of each local municipality can be found in S2 Table (Fig 1). A local municipality is a political subdivision of a province within which a municipal corporation provides local government for a specific population concentration in a defined area. Each local municipality comprises of a different number of wards; wards are geopolitical subdivisions of municipalities used for electoral purposes typically comprised of a few streets (S1 Fig). Pietermaritzburg serves as the provincial and legislative capital of the district.

### Data collection

During the SARS-CoV-2 pandemic, either nasopharyngeal or oropharyngeal specimens were collected by clinicians throughout the district and sent to laboratories where they were analysed using polymerase chain reaction (PCR). Rapid antigen testing was conducted at the point of collection at facilities with access to rapid test kits. Specimens were collected for symptomatic clients, patients prior to admission and close contacts of confirmed cases depending on the testing protocol. This occurred in the private and public sectors. Data of SARS-CoV-2 positive cases were collated, in the form of line lists, daily by the National Institute for Communicable Diseases (NICD) and channelled to the provincial health office which then decentralized cases to the district health offices daily based on geographical residence of cases. Data of laboratory-confirmed SARS-CoV-2 cases, including demographic data, i.e., age, gender, specimen collection date, local municipality name and ward number were obtained from the NICD daily line lists.

### Data analysis

Descriptive analysis was conducted separately for each of the four waves. Data were stratified by Local municipality of residence and missing values denoted as “missing” with the frequency of missing values being reported. The proportion for each independent variable (Age group, gender and Municipality) was assessed according to whether the observed differences were statistically significant at the 5% level using the Analysis of Variance (ANOVA) test, reporting the associated p-value. Statistical analyses were conducted using Stata statistical software for data analysis and programming.

In implementing temporal analysis, time series analysis using the Python Seaborn library was used, with separate modelling carried out for each wave. Specimen collection date was used to complete temporal analysis.

Spatial distribution patterns of SARS-CoV-2 were analysed at ward and municipal levels. Base layers for the district used to complete the analysis were obtained from the municipal demarcation board which is an open data source (S3 Table).

To analyse the spatial distribution of SARS-CoV-2 we used a variety of spatial methods. Kernel density estimates were used to capture where the number of SARS-CoV-2 cases was highest within the study area. Global and Local spatial autocorrelation methods were used to determine if there was clustering of SARS-COV-2 incidence rates and where statistically significant clusters and hotspots were located. All analyses were performed using data available at the ward level.

KDE (Kernel Density Estimation) was used to map density of cases over the 4 waves. KDE was conducted using the total number of positive cases for each ward, where each ward was represented by the centroid of that ward. The centroid for each ward was calculated in ArcGIS Pro by determining the most central point of each polygon. For each SARS-COV-2 wave, a KDE analysis was conducted using a 5 km search radius. The kernel function is based on the quartic kernel function [8,9].

Global Moran’s I and LISA (local indicators of spatial association) analyses were used to assess clustering of SARS-CoV-2 incidence rates. The Global Moran’s I spatial statistic was used to assess clustering for the study area. Values range from − 1 to + 1 with no spatial autocorrelation for values of 0 [10]. A positive value for Moran’s I indicates that a feature has neighbouring features with similarly high or low values (positive spatial autocorrelation) while a negative Moran’s I value indicates that a feature has neighbouring features with dissimilar values and is an outlier [11].

The Anselin Local Moran’s I (Local Indicators of Spatial Association (LISA)) test statistic [10] was used to identify where clusters were located, and their statistical significance was used. Positive spatial autocorrelation or disease hotspots were found where neighbouring locations with high SARS-CoV-2 incidence rates were found (e.g., high incidence rate locations surrounded by locations with high incidence rates (HH)). Low incidence rates of disease occurrence were identified as low-low (LL) areas. In contrast, negative spatial autocorrelation or spatial outliers occur where dissimilar values occur at neighbouring locations. These are referred to as high-low (HL) and low-high (LH) spatial outliers respectively and occur when areas of high incidence rates are surrounded by areas of low incidence rates and vice-versa [11].

All analyses were performed using ESRI ArcGIS Pro v2.0.

## Results

Statistically significant differences in SARS-CoV-2 incidence rates were observed across local municipalities and age groups. The central region of the district experienced a higher level of clusters and densities indicated by the LISA and KDE related to incident cases and incidence rates. Descriptive temporal analysis indicated fluctuation of daily positive cases at the beginning of each wave with less fluctuation in identified cases within the middle to end of each wave.

### Description of SARS-CoV-2 infection

Table 1 data obtained from NICD daily SARS-CoV-2-line lists per wave.

**Table 1:** The duration and number of SARS-CoV-2 incident cases per wave, uMgungundlovu district.

Wave	Duration	Number of laboratory confirmed SARS-CoV-2 cases
Total	–	58,797
1	23/06/2020 to 30/08/2020	10,304
2	07/12/2020 to 06/03/2021	17,159
3	09/06/2021 to 25/09/2021	20,765
4	30/11/2021 to 23/01/2022	10,569

Population size data reflected in Table 2 was obtained from Wazimap using census 2011 and community survey 2016 data [12].

**Table 2:** Population size per local municipality by age group and gender, uMgungundlovu district.

Category	District Population size	Msunduzi	uMngeni	Impendle	Richmond	uMkhambathini	uMshwathi	Mpofana
Total population size per Local municipality	–	618 536	92 710	30 382	65 540	54 933	106 388	34 913
Age group								
0-9	248 617	147 773	21 909	8 581	19 973	12 992	28 462	8 926
10-19	214 836	126 390	21 094	7 423	15 424	11 826	25 419	7 261
20-29	214 463	138 754	20 391	4 380	12 725	11 427	19 353	7 432
30-39	160 557	106 498	16 083	2 673	8 454	8 005	13 365	5 479
40-49	107 764	68 871	12 002	2 203	6 314	5 066	9 396	3 910
50-59	73 783	45 956	7 817	1 725	4 238	3 898	7 744	2 406
60-69	49 768	30 064	5 633	1 665	2 857	2 704	5 428	1 417
70-79	19 176	10 980	3 545	567	894	919	1 862	410
80+	6897	3 753	1 392	309	440	239	616	151
Gender								
Male	528 727	296 897	44 501	14 887	32 115	26 917	51 066	16 409
Female	567 138	321 639	48 209	15 495	33 425	28 016	55 322	18 504

Incidence rates reflected in Tables 3–6 were calculated using population data reflected in Table 2.

**Table 3:** Descriptive analysis of SARS-CoV-2 cases, wave 1.

Variables	Impendle	Mpofana	Richmond	uMngeni	uMshwathi	Mkhambathini	Msunduzi	Significance
**Municipal Average Incidence Rate**	47.56¹ (96)²	147.20 (204)	79.27 (369)	369.81(733)	44.84 (263)	34.71 (138)	230.61 (8501)	44.59³ (0.0000)⁴
**Gender (Female)**	40.66 (63)	65.39 (121)	67.91 (227)	94.17 (454)	30.01 (166)	31.05 (87)	162.98 (5242)	70.376 (0.0000)
**Gender (Male)**	22.17 (33)	50.58 (83)	44.22 (142)	62.70 (279)	19.00 (97)	18.95 (51)	109.77 (3259)	
**0-9 Years**	49.30 (4)	164.82 (6)	72.95 (8)	427.69 (23)	55.03 (5)	32.41 (9)	212.73 (298)	7.72 (0.0000)
**10-19 Years**	32.64 (15)	157.29 (18)	71.75 (63)	328.83 (60)	37.47 (21)	31.25 (14)	230.43 (517)	
**20-29 Years**	43.24 (14)	139.62 (55)	83.05 (70)	382.46 (95)	45.72 (32)	38.46 (20)	227.06 (1301)	
**30-39 Years**	60.08 (28)	145.37 (57)	82.70 (89)	374.05 (181)	45.99 (66)	33.43 (29)	217.75 (2132)	
**40-49 Years**	46.88 (15)	150.75 (33)	74.84 (67)	332.71 (153)	45.81 (60)	35.51 (35)	228.54 (1768)	
**50-59 Years**	50.60 (9)	147.66 (13)	90.86 (46)	378.53 (112)	42.94 (46)	35.35 (15)	246.93 (1302)	
**60-69 Years**	47.22 (6)	157.18 (13)	79.79 (15)	357.17 (65)	42.42 (20)	35.61 (10)	243.31 (706)	
**70-79 Years**	37.09 (3)	136.82 (7)	57.18 (9)	414.97 (24)	55.44 (9)	34.78 (5)	253.57 (155)	
**80 + Years**	12.67 (1)	173.84 (1)	34.13 (2)	539.62 (19)	47.14 (2)	19.11 (1)	258.28 (155)	

¹ average incidence rate calculated for subgroups per 10 000 population.

² frequency or number of subgroup participants.

³ ANOVA test, F statistic.

⁴ P-value, F Statistic.

**Table 4:** Descriptive analysis of SARS-CoV-2 cases, wave 2.

Variables	Impendle	Mpofana	Richmond	uMngeni	uMshwathi	Mkhambathini	Msunduzi	Significance
**Municipal Average Incidence Rates**	211.60¹ (534)²	284.02 (522)	251.11 (1194)	442.90 (1042)	143.79 (764)	78.32 (339)	281.24 (12 764)	363.47³ (0.0000)⁴
**Gender (Female)**	214.91 (333)	172.40 (319)	229.47 (767)	121.35 (585)	88.39 (489)	73.53 (206)	234.86 (7554)	10 549.30 (0.0000)
**Gender (Male)**	98.74 (147)	109.70 (180)	123.62 (397)	100.22 (446)	51.11 (261)	45.70 (123)	165.55 (4915)	
**Gender (Missing)**	(54)	(23)	(30)	(11)	(14)	(10)	(295)	
**0-9 Years**	186.75 (55)	285.63 (77)	229.14 (135)	390.60 (119)	122.27 (93)	80.09 (46)	281.79 (1559)	6.87 (0.0000)
**10-19 Years**	265.21 (123)	307.95 (65)	244.17 (141)	367.40 (122)	124.26 (74)	75.31 (31)	269.73 (839)	
**20-29 Years**	203.47 (67)	287.65 (103)	250.78 (235)	397.92 (146)	138.16 (111)	75.74 (58)	283.73 (1971)	
**30-39 Years**	194.42 (59)	281.59 (112)	255.57 (270)	397.39 (158)	150.89 (124)	78.23 (69)	278.35 (2565)	
**40-49 Years**	202.06 (64)	278.37 (74)	277.94 (165)	458.46 (178)	143.90 (136)	83.23 (62)	289.68 (2402)	
**50-59 Years**	189.23 (80)	273.88 (50)	233.72 (127)	425.45 (135)	159.55 (104)	78.87 (31)	279.63 (1718)	
**60-69 Years**	199.85 (56)	306.80 (25)	242.90 (82)	530.63 (82)	138.88 (71)	74.91 (30)	273.53 (1055)	
**70-79 Years**	191.24 (30)	211.69 (9)	290.94 (37)	710.47 (87)	185.12 (49)	69.39 (8)	288.74 (452)	
**80 + Years**		172.88 (7)	149.19 (2)	329.81 (15)	74.63 (2)	83.13 (4)	274.04 (203)	

¹ average incidence rate calculated for subgroups per 10 000 population.

² frequency or number of subgroup participants.

³ ANOVA test, F statistic.

⁴ P-value, F Statistic.

**Table 5:** Descriptive analysis of SARS-CoV-2 cases, wave 3.

Variables	Impendle	Mpofana	Richmond	uMngeni	uMshwathi	Mkhambathini	Msunduzi	Significance
**Municipal Average Incidence Rate**	146.94¹ (385)²	934.95 (1064)	225.84 (1016)	1 419.26 (2223)	152.21 (594)	121.38 (452)	498.28 (15031)	1 251.54³ (0.0000)⁴
**Gender (Female)**	151.02 (234)	316.69 (586)	170.83 (571)	240.20 (1158)	68.33 (378)	91.02 (255)	266.01 (8556)	3 599.90 (0.0000)
**Gender (Male)**	97.40 (145)	286.43 (470)	134.83 (433)	237.07 (1055)	41.71 (213)	70.96 (191)	215.23 (6390)	
**Gender (Missing)**	(6)	(8)	(12)	(10)	(3)	(6)	(85)	
**0-9 Years**	156.26 (31)	932.28 (225)	240.25 (142)	1 240.19 (277)	150.42 (91)	131.55 (62)	504.54 (2117)	35.22 (0.0000)
**10-19 Years**	150.37 (220)	926.99 (245)	205.31 (370)	1 290.42 (446)	147.72 (85)	108.72 (139)	441.82 (2475)	
**20-29 Years**	147.20 (52)	875.55 (200)	220.92 (134)	1 345.57 (267)	154.30 (103)	129.42 (53)	513.70 (2475)	
**30-39 Years**	146.04 (32)	986.36 (168)	238.36 (144)	1 374.14 (387)	155.77 (101)	119.97 (70)	486.18 (2963)	
**40-49 Years**	124.56 (23)	958.62 (128)	231.72 (124)	1 468.03 (375)	149.43 (91)	129.78 (63)	526.97 (2600)	
**50-59 Years**	141.44 (12)	997.69 (64)	260.21 (56)	1 473.86 (201)	166.45 (62)	126.03 (35)	501.75 (1423)	
**60-69 Years**	113.24 (10)	854.91 (23)	280.26 (26)	1 669.29 (121)	158.94 (34)	122.82 (22)	549.22 (563)	
**70-79 Years**	149.80 (2)	971.26 (10)	280.82 (13)	2 049.57 (141)	116.27 (23)	127.42 (5)	582.76 (327)	
**80 + Years**	108.08 (3)	1 165.57 (1)	171.93 (7)	896.35 (8)	135.99 (4)	138.06 (3)	373.02 (98)	

¹ average incidence rate calculated for subgroups per 10 000 population.

² frequency or number of subgroup participants.

³ ANOVA test, F statistic.

⁴ P-value, F Statistic.

**Table 6:** Descriptive analysis of SARS-CoV-2 cases, wave 4.

Variables	Impendle	Mpofana	Richmond	uMngeni	uMshwathi	Mkhambathini	Msunduzi	Significance
**Municipal Average Incidence Rate**	42.47¹ (104)²	299.39 (347)	79.43 (375)	1216.48 (1630)	71.29 (305)	65.41 (187)	277.29 (7621)	1 495.65³ (0.0000)⁴
**Gender (Female)**	43.89 (68)	103.76 (192)	65.52 (219)	185.86 (896)	35.25 (195)	38.19 (107)	137.98 (4438)	2 069.94 (0.0000)
**Gender (Male)**	21.50 (32)	73.13 (120)	47.02 (151)	160.00 (712)	19.38 (99)	27.49 (74)	101.95 (3027)	
**Gender (Missing)**	(4)	(35)	(5)	(22)	(11)	(6)	(156)	
**0-9 Years**	46.38 (9)	281.44 (62)	92.06 (50)	1 010.41 (179)	69.33 (35)	71.44 (30)	311.26 (858)	27.92 (0.0000)
**10-19 Years**	44.89 (14)	312.28 (32)	71.30 (36)	1 190.92 (171)	76.93 (24)	69.68 (23)	301.40 (648)	
**20-29 Years**	46.19 (17)	294.74 (78)	78.35 (77)	1 167.98 (251)	72.94 (58)	61.95 (31)	262.02 (1437)	
**30-39 Years**	40.24 (22)	287.01 (75)	74.47 (80)	1 143.23 (302)	71.27 (71)	65.55 (47)	255.11 (1732)	
**40-49 Years**	43.32 (16)	327.96 (47)	82.33 (67)	1 183.83 (251)	68.55 (58)	62.72 (29)	272.01 (1395)	
**50-59 Years**	39.20 (11)	305.98 (26)	90.79 (31)	1 156.85 (184)	71.52 (28)	67.05 (10)	297.31 (792)	
**60-69 Years**	44.48 (6)	319.18 (17)	81.52 (18)	1 532.79 (158)	70.20 (19)	62.11 (13)	293.89 (464)	
**70-79 Years**	40.10 (4)	319.93 (6)	53.98 (15)	1 572.47 (128)	72.29 (9)	47.18 (2)	284.64 (248)	
**80-100 Years**	29.79 (5)	303.76 (4)	18.31 (1)	1 079.06 (6)	71.87 (3)	47.18 (2)	225.15 (47)	

¹ average incidence rate calculated for subgroups per 10 000 population.

² frequency or number of subgroup participants.

³ ANOVA test, F statistic.

⁴ P-value, F Statistic.

During wave 1, 10 304 laboratory confirmed incident cases were reported as shown in Table 3. The Wave 1 analysis of SARS-CoV-2 incidence rates across the seven municipalities show significant disparities in infection rates, both spatially and across population subgroups. Incidence rates are measured per 10 000 population and disaggregated by gender and age group.. The analysis is statistically supported by ANOVA F-tests, with significance established at p-values below 0.001. The average municipal incidence rate shows substantial variability, with uMngeni reporting the highest average incidence at 369.81 per 10 000, followed by Msunduzi (230.61 per 10 000) and Mpofana (147.20 per 10 000). On the opposite end, Mkhambathini (34.71), uMshwathi (44.84), and Impendle (47.56) recorded the lowest average rates. Richmond, at 79.27, falls in the lower-middle range. (p = 0.0000). Gendered patterns in the data reveal consistently higher incidence rates among females compared to males in all municipalities (p = 0.0000). Incidence rates increase with age, with older cohorts exhibiting higher incidence rates. Among all age categories, the incidence rate was highest in uMngeni, significantly higher than in other municipalities. In contrast, Mkhambathini and uMshwathi maintained consistently lower incidence rates across all age brackets. The overall trend reveals a strong and statistically significant relationship between age and incidence (F-statistic = 7.72; p = 0.0000), with increasing age associated with higher SARS-CoV-2 incidence rates.

There were 17 159 laboratory confirmed incident cases in wave 2 (Table 4). The municipality of uMngeni reported the highest incidence rate of 442.90 per 10 000, followed by Mpofana (284.02 per 10 000), Msunduzi (281.24 per 10 000), and Richmond (251.11 per 10 000). Conversely, the lowest incidence rates were observed in Mkhambathini (78.32 per 10 000), uMshwathi (143.79 per 10 000), and Impendle (211.60 per 10 000) (p = 0.0000). This strongly suggests that the observed differences in SARS-CoV-2 incidence rates across municipalities are not due to random chance but reflect genuine geographical disparities. Gender-disaggregated analysis further indicate a consistent pattern across all municipalities: females had significantly higher incidence rates than males (p = 0.0000). Age-specific decomposition of incidence rates reveals a clear age gradient in incidence rates, with older age groups experiencing significantly higher rates of infection, particularly in the uMngeni municipality (p = 0.0000). The contrast between uMngeni and Mkhambathini also suggests that structural or systemic differences at the municipal level influence the spread and detection of SARS-CoV-2 across age cohorts. Results highlight the significant spatial and demographic inequalities in the burden of SARS-CoV-2 within uMgungundlovu district.

In wave 3 (Table 5), the aggregate number of laboratory confirmed incident cases was 20 765. The third wave of the SARS-CoV-2 presented a markedly different distribution of incidence rates across municipalities, genders, and age categories compared to previous waves. The municipality of uMngeni recorded the highest average incidence rate at 1 419.26 per 10 000 population, followed closely by Mpofana (934.95 per 10 000) and Msunduzi (498.28 per 10 000). These high rates contrast sharply with the lower rates observed in Mkhambathini (121.38 per 10 000), uMshwathi (152.21 per 10 000), and Impendle (146.94 per 10 000). Richmond showed a moderate incidence rate of 225.84 per 10 000 (p = 0.0000). In all municipalities, females experienced higher incidence rates than males (F = 3,599.90; p = 0.0000). Across nearly all municipalities, the 0–9 and 10–19-year age categories show relatively higher incidence rates. This suggests a shift toward increased infection in younger populations during the third wave. However, uMngeni recorded the highest rates for individuals aged 60–69 years (1 669.29 per 10 000), 70–79 years (2 049.57 per 10 000). Similarly, Mpofana displayed elevated incidence among elderly populations, with the 80 + year age group recording 1 165.57 per 10 000. Mkhambathini and uMshwathi continued to report lower incidence rates across all age brackets (p = 0.0000).

In wave 4, the total number of laboratory confirmed incident cases were 10 569 as shown in Table 6. uMngeni reported an incidence rate of 1 216.48 per 10 000, significantly higher than other municipalities. This was followed by Mpofana (299.39 per 10 000), Msunduzi (277.29 per 10 000), and Richmond (79.43 per 10 000). In contrast, Impendle (42.47 per 10 000), uMshwathi (71.29 per 10 000), and Mkhambathini (65.41 per 10 000) reported considerably lower average incidence rates (p = 0.0000)*.* Gender disaggregation showed statistically significant higher incidence rates in females across all municipalities (p = 0.0000). Age-based incidence patterns show a well-defined age gradient, with older age groups continuing to experience the highest rates of infection, while noting increased incidence rates in younger age groups compared to wave 1. uMngeni displayed an especially steep age-incidence profile, with incidence rates increasing from younger to older age groups. Even among children aged 0–9 years, uMngeni had a high incidence rate of 1 010.41 per 10 000. Across nearly all age groups, Mpofana also demonstrated elevated incidence rates, particularly among those aged 10–19 years (312.28 per 10 000) and 60–69 years (319.18 per 10 000). Mkhambathini and uMshwathi maintained consistently lower incidence rates across all age groups (p = 0.0000).

### Temporal analysis of SARS-CoV-2 cases per wave

**Fig 2:**
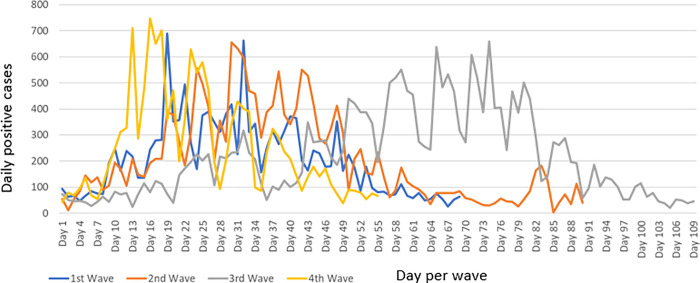
Comparison of daily laboratory confirmed SARS-CoV-2 incident cases per wave, uMgungundlovu district.

Fig 2 represents daily laboratory confirmed incident cases per wave, from the first day of the wave to the last day of the wave. Differences in the duration per wave, time taken to reach the peak of each wave as well as fluctuations in daily cases are represented. Waves 1 and 4 were shorter in duration, although wave 4 recorded the highest daily cases. Wave 2 and 3 showed a noticeably increased duration with wave 3 showing marked daily fluctuations in cases and a longer duration before reaching the peak.

**Fig 3:**
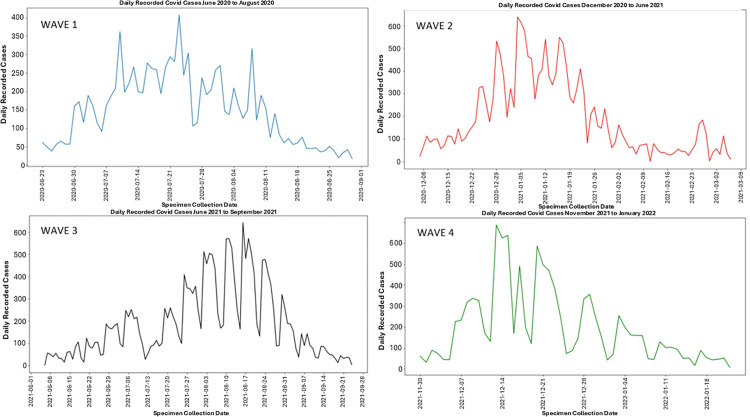
Time series plot of laboratory confirmed SARS-CoV-2 incident cases, uMgungundlovu district.

In wave 1 (Figs 2 and 3), the time series plot shows incident cases based on tests conducted from June 23, 2020, to August 30, 2020. The median and range of cases is reflected in Table 7. Cases are seen to increase to a peak with a slightly more gradual reduction in cases towards the end of the period. From late June 2020, the number of positive cases were low, but a significant increase in positive cases was noted with the onset of July, which decreased towards the end of July. The largest positive cases were recorded during July, with large daily fluctuations observed. In South Africa, during the first wave, the predominant variant (>90% of viral sequences) was the ancestral strain with an Asp614Gly mutation (June to August 2020) [3].

**Table 7:** Median and range of daily laboratory confirmed SARS-CoV-2 incident cases per wave.

Wave	Median	Range
1	179	26–690
2	148	3–655
3	192	21–659
4	195	38–747

In wave 2 (Figs 2 and 3), the time series plot shows incident cases based on tests conducted from December 7, 2020, to March 6, 2021. The median and range of cases is reflected in Table 7. During this wave, positive cases increase rapidly to a peak with a more gradual reduction towards the end of the period. Incident cases can be observed to have fluctuated in the months between December 2020 and February 2021. A drop in the incident cases is observed in mid-February, with fluctuations in daily cases decreasing as the wave transitions towards March 2021. Very high positive tests of up to 600 cases per day were recorded for the period between January 2021 and February 1, 2021; when compared to daily positive tests for the duration of the wave. The predominant variant was the Beta variant [3].

In wave 3 (Figs 2 and 3), the time series plot shows incident cases for the period June 9, 2021, to September 25, 2021. The median and range of cases is reflected in Table 7. There was a gradual increase in cases to a peak with a more rapid decline in cases towards the end of the period. Higher daily incident cases are noted towards the middle of the period with daily fluctuation, within the beginning of the period lower daily incident case fluctuations are noted. The end of the period shows decreasing cases. In South Africa, during the third wave, the predominant variant was the Delta variant [3].

In wave 4 (Figs 2 and 3), the time series plot shows incident cases for the period November 30, 2021, to January 23, 2022. The median and range of cases is reflected in Table 7. The wave is seen to peak early with large daily fluctuations and a more gradual decline in cases, fluctuations decrease towards the end of the period. The fourth wave is much shorter than previous waves. In South Africa, during the fourth wave, Omicron was the predominant variant [3].

### Spatial analysis of SARS-CoV-2 infection

**Fig 4:**
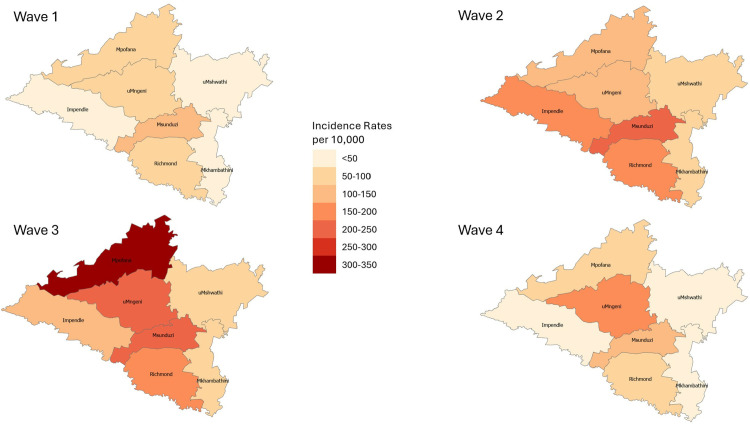
Choropleth map of SARS-CoV-2 incidence rates per local municipality, per wave, uMgungundlovu district.

Fig 4 illustrates SARS-CoV-2 incidence rates per local municipality. Generally, the central portion of the district was affected by higher incidence rates; however, in wave 3, Mpofana local municipality experienced a noticeable spike in cases.

**Fig 5:**
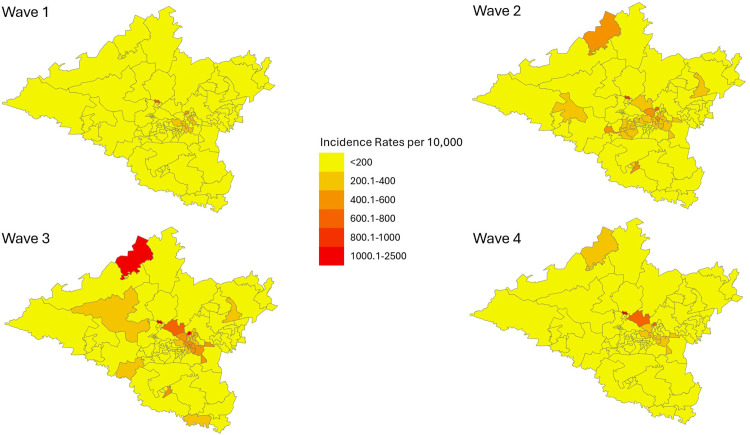
Choropleth map of SARS-CoV-2 incidence rates per ward, per wave, uMgungundlovu district.

Fig 5 illustrates incidence rates of SARS-CoV-2 per ward. Within each local municipality, the wards that experienced higher incidence rates were the wards with higher economic activity. These included: Impendle ward 4; Richmond ward 1; uMngeni ward 2; Mkhambathini ward 4; Mpofana ward 1; Msunduzi wards 24, 25, 26, 27, 28, 33, 34, 35, 36, 37 and uMshwathi ward 2.

### Kernel density estimation (KDE)

**Fig 6:**
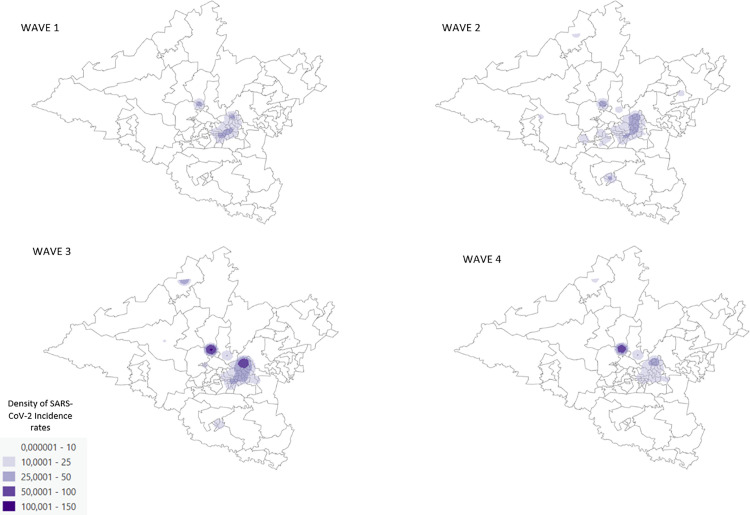
SARS-CoV-2 incident case density maps per wave, uMgungundlovu district.

Areas of higher density per wave are nearly identical (Fig 6). The central region of the district characterised by higher economic activity, population density and a built environment experienced the highest densities indicating that the risk of exposure to the virus was the highest in these regions.

### Global Moran’s I and Local indicators of spatial association (LISA)

The spatial distribution of SARS-CoV-2 varied with each Wave. During Wave 1 and 3 the spatial distribution exhibited clustering while during Waves 2 and 4 these were randomly distributed (Table 8).

**Table 8:** Global Moran’s I results for waves 1, 2, 3 and 4, uMgungundlovu district.

	Wave 1	Wave 2	Wave 3	Wave 4
**Moran’s I**	0.096	0.023	0.039	0.023
**Z-Score**	3.967	1.234	1.969	1.479
**p-value**	0.000073	0.217	0.049	0.139
**Spatial Pattern**	Clustered	Random	Clustered	Random

In all waves, the data is skewed to the right with more wards having a lower frequency of cases and fewer wards having a high frequency of cases (wave 1: median = 45.5; wave 2: median = 141.5; wave 3: median = 123.0; wave 4: median = 55.5).

**Fig 7:**
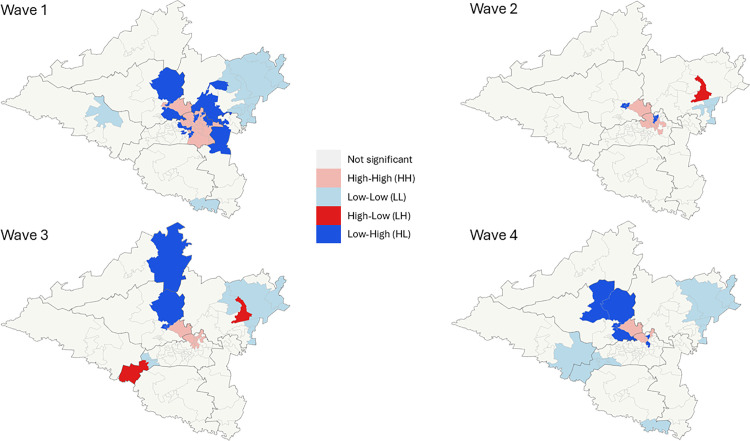
LISA analysis of SARS-CoV-2 incident cases per wave, uMgungundlovu district.

Across all four waves (Fig 7) HH clusters of statistically significant incidence rates were concentrated in the central wards of the district affecting northern Msunduzi and southern uMngeni. LL Clusters were found on the outskirts of the district affecting mostly uMshwathi, Richmond and Impendle. Outliers (LH, HL) were identified throughout.

## Discussion

Higher incidence rates were noted in females, urban areas, and older age groups. Lower incidence rates were noted in children. These findings may be due to under-reporting, predominant circulating variant per wave, health seeking behaviour and immune related response. Temporal analysis showed differences in duration per wave, time taken to reach the peak of each wave as well as fluctuation in daily cases; these findings have been attributed to the predominant circulating variants per wave. There is a clear spatial pattern of the spread of the virus within the district. The highest density of confirmed cases and incidence rates of SARS-CoV-2 occurred in the central town areas of each local municipality with a marked increase in the town areas of Msunduzi, uMngeni and Mpofana local municipalities. In terms of spatial epidemiology, the built environment of higher economic activity and higher population density is highlighted as a strong contributor to increased infection and a higher likelihood of contracting the virus. These are important indicators of priority areas and population groups to target when conducting public health interventions; similar findings were reflected in the literature [13,14].

### Under-reporting of incident cases

A higher incidence rate is noted among females. This could be explained by greater health seeking behaviour [15], as such a higher proportion of women could have presented for testing, this may indicate under-reporting in males. Additionally, more women (50.8%) work in the informal sector as compared to men (49.2%) [16] which could expose women to increased close contact. Incidence rates were higher in older age groups; although this could be because of lower innate immunity, older groups are more likely to be chronic patients that need to attend healthcare facilities for services, resulting in increased testing. Considering the relatively young study population (median age 23) incidence rates in older groups could be inflated due to smaller population size. Incidence rates were lower in younger age groups, this could be due to higher innate immunity, younger community members could have experienced higher rates of asymptomatic infection, reduced testing and resulting in under-reporting. Hesitancy in community testing could also contribute to under-reporting. This was likely due to community stigma related to testing positive [17] and fear of being hospitalized without family contact as visitation was limited. This is evidenced by the high number of home deaths and high number of deaths with unknown cause of death during the pandemic period compared to previous years, therefore, temporally highlighting SARS-CoV-2 infection as a possible cause [18,19]. Geographically, uMgungundlovu district consists of urban, semi-urban, rural, and deep rural areas; although public health efforts aim to reach all areas, potentially increased testing in areas with easier access could contribute to under-reporting of cases in harder to reach areas.

### Age distribution of SARS-CoV-2 infection

In the first two waves, the highest incident cases were reported among the adult population within the age groups: 20–29 years, 30–39 years, 40–49 years, and 50–59 years. In the fourth wave incident cases were the highest among the age groups: 20–29 years, 30–39 years and 40–49 years. These age groups are generally the most mobile due to work, school, and leisure. It may be reasonable to hypothesize that this could be a reason for increased infection in these groups. It is also important to note that the population in question is a young population which may account for increased infection in younger age groups, these findings are mirrored in a study conducted by de Souza et. al. (2021) on Brazilian populations with characteristics like the study population [20]. In wave 3 the highest incident cases were recorded in the 0–9 years, 10–19 years, 20–29 years, 30–39 years, and 40–49 years age groups. This was a deviation from the first two waves with an increase in cases in younger age groups, the main explanation for this deviation was the circulating variant of the time causing a higher rate of severe disease in younger age groups [3].

Incidence rates increased across the first three waves in the 0–59-year age groups, however, incidence rates in the 60 years and older age groups decreased from wave 2 to wave 3. Before the third wave, vaccination efforts commenced within the district, however, the initial rollout of vaccines prioritised healthcare workers and high-risk population groups (the elderly and those living with underlying medical conditions) [21]. The increasing proportion of new infections in younger people has been equated to higher vaccination coverage in older population groups [22]. At the beginning of the pandemic, older and younger people engaged in similar preventive personal behaviours when controlling for other influences; however, as the pandemic progressed, older people adopted behavioural changes associated with reduced transmission more than younger people [23]. Additionally, the variant of concern per wave could have contributed to the increase in paediatric cases as the delta variant is associated with an increased disease severity and an increase in infection and symptom manifestation in a greater proportion of younger people (age 0–19 years) than earlier variants [3]. All age group related incidence rates reduced from wave three to wave four. Although incidence rates in the 0–29 years age groups remained higher in the fourth wave than wave one. This could be due to vaccination of older age groups and healthcare workers and the omicron variant which showed higher propagation rates among children than pre-Omicron waves [24]. The virus showed clear age dependent pathology, with most adults and children experiencing symptoms resembling the common cold while causing more severe respiratory distress and significant mortality in older and frail humans [3] resulting in higher testing and diagnosis of cases in older population groups. Loss of immune function and reduced protection from infectious agents with age are important explanatory factors.

### Spatial and temporal analysis

These findings add to a growing body of work examining population-level SARS-CoV-2 infection, as well as differences in infection across regions [6,9,13,14,20]. The results of the first four waves observed have clearly illustrated that the central region of the district was the most severely affected by SARS-CoV-2 incidence rates. In wave 1 and 4 the central areas of the district were surrounded by local municipalities with significantly lower incidence rates. This is interesting since according to available research, regions adjacent to areas of high transmission experience subsequent high transmission due to ‘spatial spill-over’ [25].

Within uMgungundlovu district, the first wave saw strict community level, household contact tracing and PCR testing of all close contacts, coupled with lockdown measures and fear of the unknown, most residents were willingly compliant. South Africa also experienced the first wave two months later than other countries thus benefitting from learning from other countries’ experiences [26]. These are likely contributing factors to the relatively less severe situation in the country and the district during the first wave compared to the global situation at the time [27].

In the second wave of the pandemic the infection rate was higher leading to an increase in the incidence rates of SARS-CoV-2 during this period [28]. Cases increased significantly in the second wave when the National Corona Virus Command Centre shifted the lockdown to alert level 3 [29]. This allowed some degree of mobility in the country and among communities, increased interaction created a positive environment for rapid transmission [30]. Additionally, eThekwini district, adjacent to uMgungundlovu experienced the highest incidence rate of SARS-CoV-2 due to an increase in social activities that brought a multitude of people together, resulting in ‘super-spreader’ events. This increase in contact among community members increased transmission of the virus. Additionally, incidence rates in the district of Harry Gwala which borders both Richmond and Impendle local municipalities was noted to increase approximately 2 weeks before incidence rates in Richmond and Impendle, this could be evidence of ‘spatial spill-over’ [31].

The transmission of SARS-CoV-2 and the resulting case load was much higher in the 3rd wave. This, among other explanatory reasons, could have been due to the social unrest that occurred in the district. The widespread unrest leading to massive damage to property and infrastructure was catalytic to widespread transmission in the area [5]. It is believed that this social upheaval that undermined public health preventive measures was as a results of lockdown fatigue, and slow vaccine rollout [32]. Mpofana local municipality experienced the highest incidence rate. This may be due to a relaxation on restrictions involving international trade as fresh produce from these areas are exported. Additionally, an increase in congregate setting cases particularly in boarding schools, were noted.

During wave 4, SARS-CoV-2 incidence rates looked different from the first three waves. The wave was shorter, and the cases spiked quicker reaching a peak within a much shorter time as opposed to what was observed with the initial 3 waves. This was likely due to the predominant variant – Omicron.

The LISA analysis undertaken showed that the distribution of incident cases and incidence rates of SARS-CoV-2 in uMgungundlovu were clustered in wave 1 and 3. An increase in clusters were noted in northern Msunduzi which is the central business district (CBD) of Pietermaritzburg (provincial capital) which is an area of high economic activity, population density and the built environment. Wards with statistically significant LL clustering remained on the rural outskirts of the district. This finding brings forth the built environment of higher economic activity and close contact as catalytic to infectious disease spread. Additionally, potential reduced access to healthcare services would come into question considering this finding.

The Kernel density estimation showed that areas with the highest density of confirmed SARS-CoV-2 cases occurred in the central town areas of Msunduzi, uMngeni and Mpofana local municipalities and, to a lesser extent, the central town areas of the rest of the local municipalities. Indicating that the probability of contact with the virus was higher in high density areas. Clearly lower densities were noted in rural areas. This might be an indication of increased contact within areas of higher economic activity as well as population density, contributing to increased infection. This may also be an indication of reduced access to testing facilities in rural areas, resulting in a possible underestimation of the actual burden of the COVID-19 pandemic.

## Conclusion

This is the only study to date to document the spatial distribution of SARS-CoV-2 incident cases and incidence rates in uMgungundlovu district, KwaZulu-Natal, South Africa. The observed higher concentration of incidence rates observed in the northern regions of Msunduzi and southern regions of uMngeni, might show a clear relationship between infection transmission and increased economic activity, population density and urban development. The data from this study shows that infection rates across the district were not uniform and were driven by increased community mixing in urban areas and congregate settings. Additionally, differences in infection across waves also highlight important characteristics of the predominant circulating variant, this provides useful information on the evolution of the virus. Further research could identify and explain the association between urban living and infectious disease epidemics and explore feasible and easy to implement public health interventions that could curtail a rising epidemic with existing health system structures.

## Supporting information

S1 Fig a) uMgungundlovu ward distribution b) uMngeni local municipality ward distribution and c) The Msunduzi local municipality ward distribution. Boundary data are from esri (S3 Table) and background map provided by Esri (Esri South Africa, TomTom, Garmin, FAO, METI/NASA, USGS, CGIAR). Maps were created using ArcGIS Pro software by Esri.
(TIF)

S2 Table Characteristics of local municipalities within uMgungundlovu District, KwaZulu-Natal [33]. Extracted from https://municipalities.co.za/map/120/umgungundlovu-district-municipality.
(PDF)

S3 Table Base layer data sources. (PDF)
