# Supplementary figures and images for: Spatial and temporal patterns of SARS-CoV-2 infection in uMgungundlovu, KwaZulu-Natal, South Africa
Source: PLoS One. 2026 Apr 15;21(4):e0317648. doi: 10.1371/journal.pone.0317648 (PMC13082583; doi:10.1371/journal.pone.0317648)

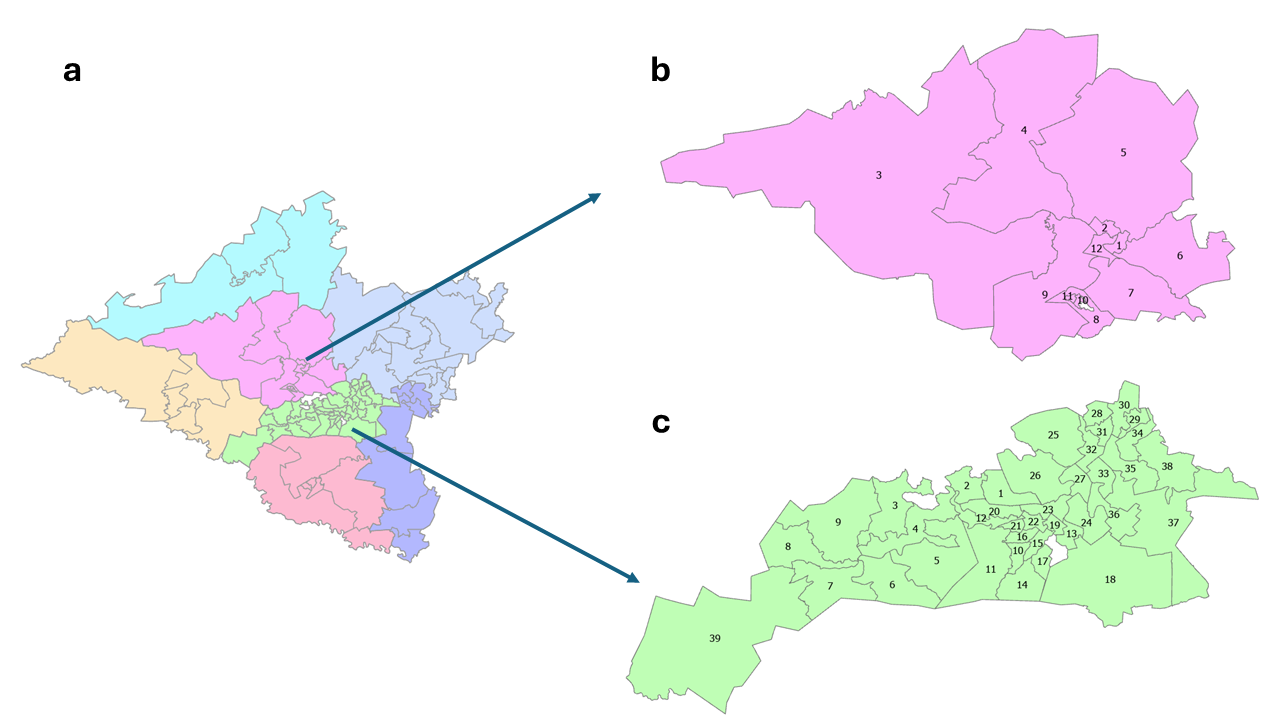

Supplement: S1 Fig — Boundary data are from esri (S3 Table) and background map provided by Esri (Esri South Africa, TomTom, Garmin, FAO, METI/NASA, USGS, CGIAR). Maps were created using ArcGIS Pro software by Esri. (TIF) [file pone.0317648.s001.tif]
