# Supplementary material for: Spatial and temporal patterns of SARS-CoV-2 infection in uMgungundlovu, KwaZulu-Natal, South Africa
Source: PLoS One. 2026 Apr 15;21(4):e0317648. doi: 10.1371/journal.pone.0317648 (PMC13082583; doi:10.1371/journal.pone.0317648)
Supplement: S2 Table — Extracted from https://municipalities.co.za/map/120/umgungundlovu-district-municipality. (PDF) [file pone.0317648.s002.pdf]

S2 Table. Characteristics of sub-districts within uMgungundlovu District – KwaZulu-Natal. Extracted from https://municipalities.co.za/map/120/umgungundlovu-district-municipality.

Sub-district	*Description*	*Population size*	*No. of wards*	*Area (km²)*	*Main economic sectors*
uMsunduzi	It is the smallest of the seven municipalities in the district. It encompasses the city of Pietermaritzburg and is densely populated due to economic activity.	618 536 (60.77%)	41	751	Community services, finance, transport, trade, manufacturing
Impendle	**S**ituated outside the primary and secondary movement systems of the district and is some distance away from the major tourist and trade routes.<br>Area is extremely diverse in its topography, climate and soil, and has a rich and complex natural environment.	33 105 (3.25%)	4	1610	Agriculture, tourism, social services
Richmond	Situated along the southern boundary of the District, approximately 38km south of Pietermaritzburg. Known for fruit and vegetable farming.	65 793 (6.46%)	7	1231	Agriculture, community services, trade, finance
uMngeni	Situated approximately 29km north-west of Pietermaritzburg. Comprises of a substantial amount of farm land.	92 710 (9.11%)	13	1521	Agriculture, wholesale/retail, business / real estate, manufacturing
uMkhambathini	Situated along the south-eastern boundary of the District. A large part of the municipality is rural and underdeveloped.	63 142 (6.20%)	7	868	Agriculture, tourism
uMshwathi	The land is mostly agricultural. The communities living in the underdeveloped areas have extremely limited access to basic physical and social requirements and very few economic opportunities.	106 374 (10.46%)	14	1866	Agriculture, manufacturing, tourism
Mpofana	Situated approximately 40km west of Pietermaritzburg. Almost 50% of the land in Mpofana has the potential for rural farming, with products set for the international market.	38 103 (3.75%)	4	1755	Agriculture, tourism
