# Supplementary material for: Spatial and temporal patterns of SARS-CoV-2 infection in uMgungundlovu, KwaZulu-Natal, South Africa
Source: PLoS One. 2026 Apr 15;21(4):e0317648. doi: 10.1371/journal.pone.0317648 (PMC13082583; doi:10.1371/journal.pone.0317648)
Supplement: S3 Table — (PDF) [file pone.0317648.s003.pdf]

**S3 Table.** Base layer data sources.

| *Data* | *Description* | *Source* | *Date accessed* | *URL* |
|---|---|---|---|---|
| *District boundaries* | GIS boundary layer | GADM | 23/06/2022 | https://gadm.org/license.html https://gadm.org/data.html |
| *Ward and municipal boundaries* | GIS boundary layer | GADM | 23/06/2022 | https://gadm.org/license.html https://gadm.org/data.html |
